# Quantification of ash sedimentation dynamics through depolarisation imaging with AshCam

**DOI:** 10.1038/s41598-018-34110-6

**Published:** 2018-10-24

**Authors:** Ben Esse, Michael Burton, Matthew Varnam, Ryunosuke Kazahaya, Paul A. Wallace, Felix Von-Aulock, Yan Lavallée, Giuseppe Salerno, Simona Scollo, Hugh Coe

**Affiliations:** 1 School of Earth and Environmental Sciences, The University of Manchester, Manchester, M13 9PL UK; 2 Geological Survey of Japan, National Institute of Advanced Industrial Science and Technology, Tsukuba, Japan; 3 Department of Earth, Ocean and Ecological Sciences, University of Liverpool, Liverpool, L69 3GP UK; 4 Istituto Nazionale di Geofisica e Vulcanologia, Osservatorio Etneo, sezione di Catania, Piazza Roma 2, 95123 Catania, Italy

## Abstract

Even modest ash-rich volcanic eruptions can severely impact a range of human activities, especially air travel. The dispersal of ash in these eruptions depends critically on aggregation and sedimentation processes – however these are difficult to quantify in volcanic plumes. Here, we image ash dynamics from mild explosive activity at Santiaguito Volcano, Guatemala, by measuring the depolarisation of scattered sunlight by non-spherical ash particles, allowing the dynamics of diffuse ash plumes to be investigated with high temporal resolution (>1 Hz). We measure the ash settling velocity downwind from the main plume, and compare it directly with ground sampled ash particles, finding good agreement with a sedimentation model based on particle size. Our new, cost-effective technique leverages existing technology, opening a new frontier of integrated ash visualisation and ground collection studies which could test models of ash coagulation and sedimentation, leading to improved ash dispersion forecasts. This will provide risk managers with improved data quality on ash location, reducing the economic and societal impacts of future ash-rich eruptions.

## Introduction

Volcanic ash is a primary product of explosive volcanism which, while often benefitting the biosphere, generally poses a threat to human health and infrastructure^1^. Ash exposure can cause irritation to the nose, throat and eyes, as well as aggravating pre-existing health conditions such as asthma^2^. Heavy ash fall can also lead to building collapse, potentially injuring or killing those inside^3^. Ash is also a danger to other critical infrastructure, including electrical, water and transportation networks (especially air travel)^4–7^. Recent work has focussed on the key role of ash aggregation in controlling the dynamics of ash plumes^8–10^, and such processes are included in new modelling approaches^11^. Testing and validation of ash aggregation and sedimentation models is therefore an urgent requirement, but we have few tools capable of providing the empirical and reliable quantification of ash dynamics in the atmosphere.

Existing ash detection methods include infrared imagery^12–14^, radar^15,16^ and LiDAR^17,18^, as well as combined analysis of acoustic signals and optical imagery^19,20^. Satellite UV instruments (such as OMI) have also been shown to be sensitive to ash^21^ – however little work has been done to date on ground based UV measurements. Two previous studies have used ground-based UV cameras for the observation of volcanic ash^22,23^, with a third measuring black carbon particles in ship emissions^24^. All three of these studies assume that absorption from the ash (or carbon particles) dominates the attenuation of the light passing through the plume and so do not explicitly detect ash. We note that for optically thick plumes reflection from the surface or internal scattering within the plume would likely dominate over transmission.

Ash particles are formed through explosive fragmentation of magma and are often very glassy in nature. They form sharp, irregular shapes that are hard to characterise morphologically^25^ and have complex interactions with radiation, leading to errors in ash mass retrievals^26^. The non-sphericity of ash particles does have one advantage however: the depolarisation of scattered light.

Here, we present a new method for investigating the dynamics of ash plumes using ground-based UV-VIS imagery named “AshCam”. By measuring the intensity of light for two orthogonally polarised channels, deviations from the expected polarisation pattern of scattered sunlight can be used to infer the presence of ash. UV-VIS cameras are widely used in volcano monitoring for measuring volcanic SO_2_ fluxes^27–30^. These systems typically use two wavelength channels, one sensitive to SO_2_ and one not, to quantify the SO_2_ column amount in each pixel. This is usually achieved with either a single camera and a filter wheel, or two cameras with two different filters. By replacing the filters with two linear polarising ones these cameras can be modified to measure ash, allowing AshCam to be easily integrated into existing monitoring networks. The high frame rate (~1 Hz) of the cameras allows for the investigation of ash dynamics, for example the ash settling velocity, and individual filaments of ash can be tracked between frames, providing detailed quantification of ash velocities.

We report on observations of mild explosive activity at Santiaguito Volcano, Guatemala. We show for the first time that it is possible to measure the depolarising effect of ash through passive measurements of sunlight and demonstrate how these measurements can be used to investigate the dynamics of ash plumes.

## Results

### Field site

**Figure 1:**
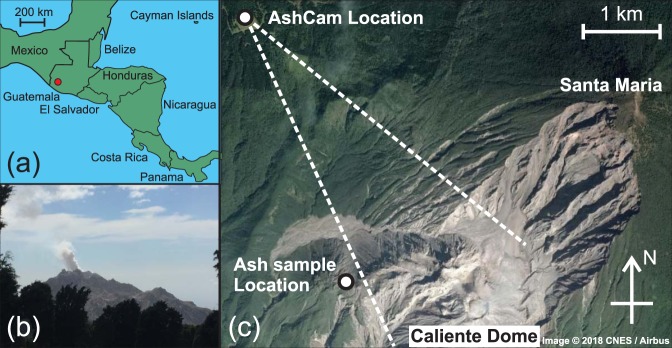
Geography of Santiaguito. (**a**) Sketch map showing the location of Santiaguito in Central America (red circle). (**b**) Image of Caliente dome taken from the measurement location at the onset of an explosion (image taken by Ben Esse). (**c**) Satellite image of the area surrounding Santiaguito with the measurement and sample locations marked. The dome complex can be seen to be growing from the collapse scar of Santa Maria. The dotted white lines give the approximate field of view of AshCam. Map data: Google, CNES/Airbus. Map generated with Google Earth version 7.1.8.3036 (https://www.google.com/earth/).

Santiaguito (14.7500° N, 91.5667° W, 2520 m) is an andesitic-dacitic lava dome complex in Guatemala (Fig. 1a) which formed after the 1902 eruption and subsequent collapse of Santa Maria^31,32^. Santiaguito exhibits regular explosions approximately once every 26 minutes to two hours from the currently active vent feeding “Caliente” dome^33,34^ (Fig. 1b). This makes it an excellent natural laboratory for testing ash remote sensing techniques^20,35^. The observations presented here were made on 18^th^ January 2018 from an observation site approximately 4 km north-west from the dome (Fig. 1c). During the observation period two explosions occurred at 09:00 and 11:10 (local time), lasting approximately 1 and 10 minutes respectively. Here, we focus on the second explosion as a test case for AshCam due to its longer duration.

### Depolarisation images

**Figure 2:**
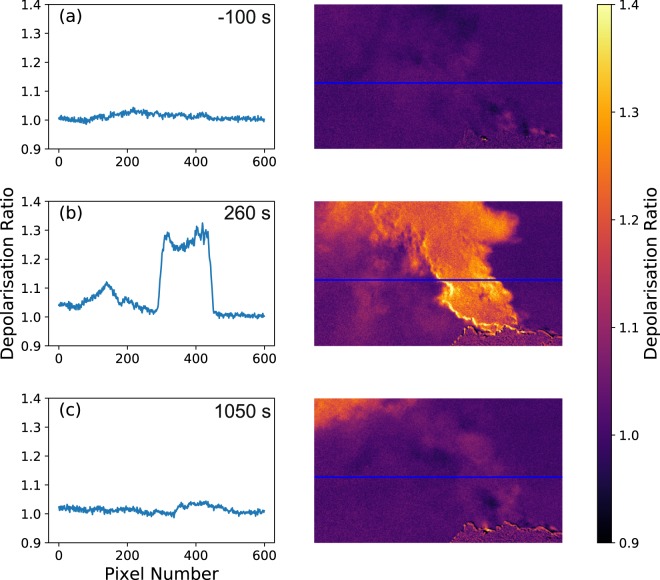
Example depolarisation images before (**a**), during (**b**) and after (**c**) an explosion at Santiaguito. The timings are relative to the onset of the explosion. The graphs depict the cross-sections indicated by the blue lines on the images. The cross-sections are averaged across 10 pixels vertically.

Figure 2 shows examples of depolarisation images taken before, during and after the explosion, as well as graphs showing the cross-section of the plume (blue line on the images). The cross-sections are averaged vertically across ten pixels. The explosion started with a smaller impulsive event, followed by a more prolonged ash emission that formed the main body of the explosion – a common feature at Santiaguito^20,34,35^. A full video of the depolarisation images for the explosion can be found in Supplementary Video S1. Images were acquired with a frequency of 0.2 Hz – although the cameras are capable of higher frame rates (5–15 Hz depending on acquisition quality settings), 0.2 Hz was chosen in the field due to data storage practicalities and to increase the image quality. The main, optically-thick aerosol plume rising from the dome has a strong depolarisation ratio (~1.25) – however this is likely due to reflections from the plume surface and multiple internal scattering within the optically thick plume itself. This means the dynamics of the ash cannot be separated from the other aerosols in the plume. We note that this result suggests direct observation of aerosol-rich plumes with SO_2_ cameras may be strongly affected by reflected sunlight. The ash settling out from the main plume could instead be readily observed as optically thin depolarisation features on the left side of the images. We highlight that the fine settling ash was not clearly visible to the naked eye or with normal video recording equipment (see Supplementary Fig. S1). The depolarisation signal (~1.1) is significantly above the background noise in the image (~1.01). The background sky shows little change between the frames, remaining close to 1 throughout the explosion. A false signal can be seen in the top left corner of frame (c) due to reflections from low meteorological clouds. Additionally, an enhancement of the signal can be seen on the edge of the main plume and on the dome due to a slight spatial misalignment of the horizontally and vertically polarised images.

### Plume dynamics

**Figure 3:**
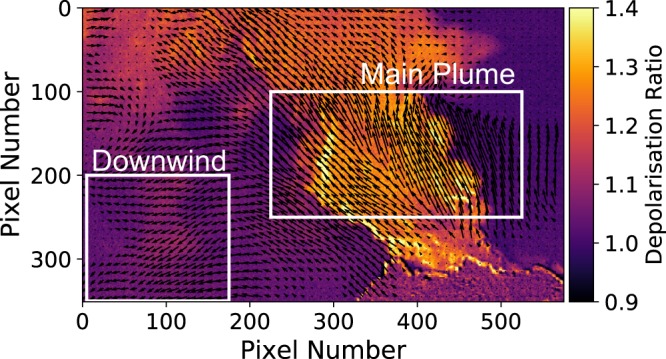
Example flow map of the output from the optical flow 275 s after the onset of the explosion. The length of the arrows is proportional to the flow speed. The white boxes show the main plume and downwind areas chosen for further analysis.

The plume dynamics were investigated using an optical flow algorithm (see methods section for details). This was achieved using the Farnebäck algorithm from the python OpenCV library. The parameters used are the same as those given in Table A.2 from Peters *et al*.^36^. Figure 3 shows an example flow field calculated by the algorithm. A full video of the optical flow output is given in Supplementary Video S1.

**Figure 4:**
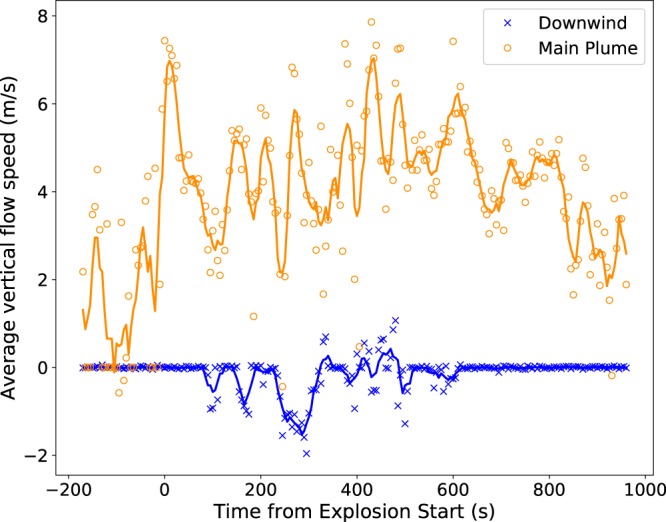
Average vertical flow speeds for the main plume (orange circles) and downwind (blue crosses) regions. The x-axis is the time with respect to the onset of the explosion, determined from the imagery. Positive velocities correspond to upwards motion. The solid lines represent the 5 point moving average.

Two regions were selected to investigate further: the main plume and the downwind settling area. The flow field is first masked by setting a threshold depolarisation level to remove the contribution of non-ash pixels, allowing the average vertical component of the ash velocity to be calculated (Fig. 4). Note that a positive velocity corresponds to upwards motion. The optical flow algorithm fails to accurately map the flow in some areas, for example immediately above the vent. It is suspected that this is due to the plume being highly turbulent when first emitted, so there are no consistent features for the optical flow algorithm to track. Additionally, the motion of meteorological clouds in the frame affects the calculated plume flow, for example in the top left corner of the frame. These regions were avoided when selecting the main plume and downwind regions.

For the main plume (Fig. 4, orange circles) the onset of the explosion can be seen in a distinct peak in velocity at time 0 s. This initial burst is then followed by a steady increase in the velocity as the explosion progresses. After 600 s the plume velocity drops off as the plume detaches from the dome. The average velocity for the first phase (0–600 s) is 4.6 m.s^−1^, while for the second (600–960 s) it is 2.7 m.s^−1^. Previous measurements at Santiaguito with infrared imagery give buoyant ascent velocities between 3.5–15.5 m.s^[−1 37^, which is in agreement with our measurements of the main plume during the explosion. There is an oscillatory pattern in the vertical velocity during the first phase with a frequency of approximately 60 seconds, perhaps reflecting a pulsatory emission pattern^35^. This shows that AshCam can still be used to investigate the rise dynamics of the main plume and eruption style, even though it is not able to separate the ash from other aerosols in optically thick plumes.

The ash settling out of the plume (Fig. 4, blue crosses) did not occur in a steady fashion, rather clumps of ash separate from the main plume and move together, suggestive of gravitational instabilities^38^. This can be seen in the three peaks between 80 and 320 s, after which the flow velocity field becomes too noisy to distinguish any significant settling. After 600 s the velocity in the downwind area returns to zero. Between 80–320 s settling velocities of 0.5–1.5 m.s^−1^ are observed. The average standard deviation of the flow speed across the three peaks is 0.4 m.s^−1^.

In addition to uncertainty in the flow speed derived by the optical flow algorithm, the measurement geometry can also introduce systematic errors. We assume that all motion is in the plane of the image, with no component either towards or away from the observer. Here the distance to the source (approximately 4 km) is large enough that this effect will be minor – however it should be considered for more proximal measurements.

### Ash particle size estimation

**Figure 5:**
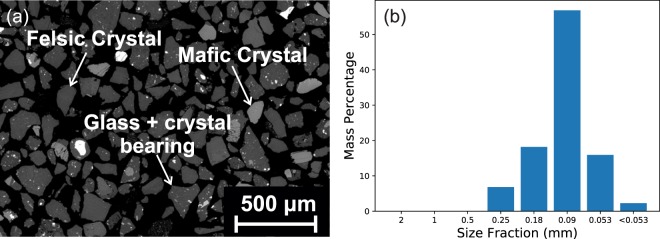
Sample ash collected from Santiaguito. (**a**) BSE SEM image of the ash sample. (**b**) Particle size distribution of the ash collected from Santiaguito on 20^th^ January 2018. The size fractions were sorted by dry-sieving the sample.

Ash settling out of the plume was collected on the 20^th^ January near to the dome (approximately 1.4 km west-northwest). Although this was a different day to when the measurements with AshCam were made, the style of activity remained constant during our observations at Santiaguito (17^th^ – 20^th^ January). This ash was dry-sieved to sort the particles into size fractions of 2 mm, 1 mm, 0.5 mm, 0.25 mm, 0.18 mm, 0.09 mm, 0.053 mm and <0.053 mm (Fig. 5). The majority of the ash (56.8% by mass) is in the 0.09 mm size fraction. The density of the ash sample was measured using 0.1 g of ash particles in a 0.1 cm^3^ sample insert within the 1 cm^3^ chamber module of a helium pycnometer (Micromeritics AccuPyc 1340), providing volumes with a precision of ±0.01% of the chamber volume. An ash density of 2679.2 kg.m^−3^ was measured (±0.2% based on 5 repeat analyses).

An estimation of the particle size can be made from the settling velocity of the ash and compared to the sample collected on the ground. The relation between the particle size and its settling velocity depends on the Reynolds Number, R_e_^39^. We use the equation for settling velocity for the intermediate Reynolds Number regime (0.4 < R_e_ < 500), as defined by Bonadonna *et al*.^39^. Given a measured settling velocity, the particle diameter, d, can be calculated:

1 $${\rm{d}}={{\rm{v}}}_{{\rm{settle}}}{({\mathrm{225}{\rm{\mu }}{\rm{\sigma }}/4{\rm{\rho }}}^{{\rm{2}}}{{\rm{g}}}^{{\rm{2}}})}^{1/3}$$

where v_settle_ is the settling velocity (m.s^−1^), μ is the dynamic viscosity of air (Pa.s), σ is the air density (kg.m^−3^), ρ is the particle density (kg.m^−3^) and g is gravitational acceleration.

We estimate the air density and viscosity to be 0.82 kg.m^−3^ and 1.84 × 10^−5^ Pa.s respectively (calculated from the altitude (3000 m) and air temperature (25 °C) at the time of the measurements). By inserting the ash density and settling velocities observed we retrieve particle diameters of between 0.05–0.16 mm, which agrees well with the particle size range found in the collected ash sample (Fig. 5b).

Care must be taken when applying particle settling models as they are for a sphere falling in a fluid. Volcanic ash is inherently non-spherical, so the true settling velocity will depend on exact shape and orientation of the particles as they fall^8^. Here, optical analysis reveals that a large fraction of the ash particles is near equant whilst a smaller population comprises elongate particles with length:width aspect ratios up ca. 3. The calculation of particle size could be further refined by taking into account the shape of the settling ash (determined from samples collected in the field), but consideration of spherical particles in the above calculation provides a first-order constraint of settling rates.

## Discussion

Recent focus on the roles of aggregation, disaggregation and sedimentation in modelling the transport of airborne ash means there is a requirement for quantification of the dynamics of airborne ash^8–11^. Robust quantification could lead to rigorous testing of ash sedimentation processes and thereby greatly improve the fidelity of modelling of near-source ash dispersal. AshCam presents a new tool to measure the dynamics of disperse ash plumes by detecting the depolarising effect of non-spherical ash particles on scattered sunlight, thereby providing a technique to quantify sedimentation processes. Ground based UV-VIS cameras are being widely used as SO_2_ cameras, and these can be easily adapted to become AshCams. We have presented observations of an ash-rich explosion at Santiaguito volcano on 18^th^ January 2018 and shown, for the first time, that it is possible to measure the depolarising effect of volcanic ash on sunlight (Fig. 2). A clear depolarisation signal can be seen both from the main plume and from settling ash downwind. The signal from the main plume cannot be attributed solely to ash due to the opaque nature of the plume, as main sources of changes in polarisation are likely to be reflections from the surface of the plume or multiple internal scattering within the column itself. In the downwind area, however, the plume is much more dispersed and so we can assume that the depolarisation is due to ash settling from the main plume. Identifying when a plume is diffuse enough for these assumptions to be valid would enable a more robust deployment of AshCam. This could perhaps be achieved using a measurement of the transmitted intensity through the plume.

We have measured the dynamics of the ash plume by using an optical flow algorithm (Fig. 3). In the main plume the average rising velocity during the explosion is 4.6 m.s^−1^ (Fig. 4, orange circles), which agrees with past measurements made at Santiaguito using infrared imagery^37^. In the downwind region the ash settling velocity is measured to be between 0.5–1.5 m.s^−1^ (Fig. 4, blue crosses), which corresponds to a particle diameter of between 0.05–0.16 mm, which agrees well with the samples collected on the ground (Fig. 5).

AshCam is a powerful new tool for volcanologists, providing a cheap and easy way in which existing instrumentation (the SO_2_ camera^27–30^) can be adapted to provide entirely new datasets. Any UV SO_2_ camera can be easily converted into AshCam by replacing the normal filters with polarising ones (see methods), meaning that AshCam can be quickly, easily and cheaply integrated into existing volcano monitoring networks. The potential impact of AshCam could be much greater than an entirely novel system, as much of additional work required to produce an operational monitoring network has already been accomplished (for example power supply, automation and data transfer^40,41^). As AshCam is a passive system it is much more portable and less expensive than many other ash detection methods, such as LiDAR or radar. This allows it to be deployed in more remote locations or in rapid response to new eruptions.

We have applied AshCam to a single explosion at Santiaguito volcano. The complex topography of Santiaguito and nearby Santa Maria meant we were unable to measure the ash settling velocity as a function of distance from the dome – however this could be applied to other volcanoes. AshCam could also be used to investigate other phenomena, such as the role of gravitational instabilities in removing fine ash from plumes^38,42^. Deployment of AshCam alongside other monitoring techniques (for example seismic data or an SO_2_ camera) would allow further investigation into volcanological processes. It would also be interesting to compare AshCam with ground based IR cameras, which are often used to image ash plume dynamics and are not affected by polarisation^13^.

The results presented here demonstrate the ability of AshCam to investigate the settling dynamics of volcanic ash at high temporal resolution. This will allow for testing the roles of aggregation and sedimentation in ash dispersal models, leading to more robust and accurate ash forecasting models and, therefore, reducing the social and economic costs of future ash-rich eruptions.

## Methods

### Measuring Depolarisation from Ash

Volcanic ash is formed through explosive fragmentation of volcanic glass and bubble walls, producing a diverse range of shapes and sizes including long sharp needles and flat plates. This heterogeneity makes modelling the optical properties of ash difficult, and so it is often assumed to be spherical in shape which can lead to errors in ash retrievals^26^. The non-spherical nature of ash does offer one advantage - the depolarisation of scattered light.

The principle of using depolarisation from non-spherical scattering aerosols was first applied to distinguish ice and water clouds in LiDAR measurements^43^. The same method has also been applied to LiDAR measurements of volcanic ash clouds to separate the ash from other aerosols^44–46^. Details of the interaction of light with non-spherical particles are outlined by Sun *et al*.^47^.

AshCam is a passive system, so the light source is not controlled as with LiDAR. As the angle of polarisation is a function of both the viewing direction and time, measuring the absolute depolarisation ratio is difficult. Instead, we measure changes in the polarisation state of the sunlight to infer the presence of ash. To achieve this, each raw image is normalised with a reference image taken before the onset of the explosion when no plume or ash is present. The horizontally polarised channel is then divided by the vertically polarised channel. All images were corrected for the dark current in the CCDs using dark images collected periodically during data acquisition. The resulting images map changes in the polarisation state of the measured light from the reference image.

This method assumes that the light is forward-scattered, not scattered at an angle. This means that for dense ash plumes, such as the main plume at Santiaguito, we cannot assume that the depolarisation signal seen is only from ash. The main source will be multiple internal scattering within the plume or reflection from its surface. In these cases AshCam can still be used to investigate the dynamics of the plume as a whole.

### Equipment

**Figure 6:**
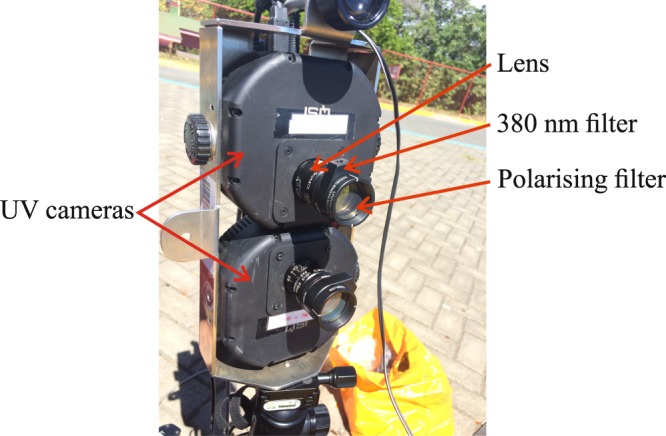
Setup of AshCam. The cameras are fitted with a 380 nm band filter (FWHM = 10 nm) and a polarising filter mounted to the front of each lens. The cameras are mounted on a standard tripod and powered using a pair of Lithium Polymer batteries.

We used two UV-VIS cameras (QSI 620 s) to detect ash settling from the plume (Fig. 6). The image dimensions are 1200 × 1600 pixels and the field of view is 26.64°. A frame rate of 0.2 Hz was used, allowing the dynamics of explosive events to be recorded. The cameras are each mounted with a 380 nm band filter (Thorlabs FB380-10 bandpass filter, FWHM = 10 nm) and a linear polarising filter (Thorlabs LPUV100-MP2) in front of the lens (RICOH FL-BC2528-VGUV). The two polarised filters are installed orthogonally to each other to measure the intensities of horizontally and vertically polarised light (with respect to the horizon). Both cameras acquire images simultaneously and are controlled with a laptop computer.

The entire equipment setup is easily carried in a backpack, making it extremely portable and well suited to measurements in remote locations.

### The Polarisation Pattern of Skylight

Rayleigh scattering of sunlight within the Earth’s atmosphere means the sky acts as a diffuse light source, appearing blue as shorter wavelengths of light are more preferentially scattered than longer ones. Although natural sunlight is not polarised, this scattering introduces a degree of linear polarisation in the observed skylight. There is strong evidence that the polarisation pattern of the skylight is used as a navigational tool by a number of insect species^48^, as well as (possibly) by Vikings navigating under cloudy conditions^49^.

**Figure 7:**
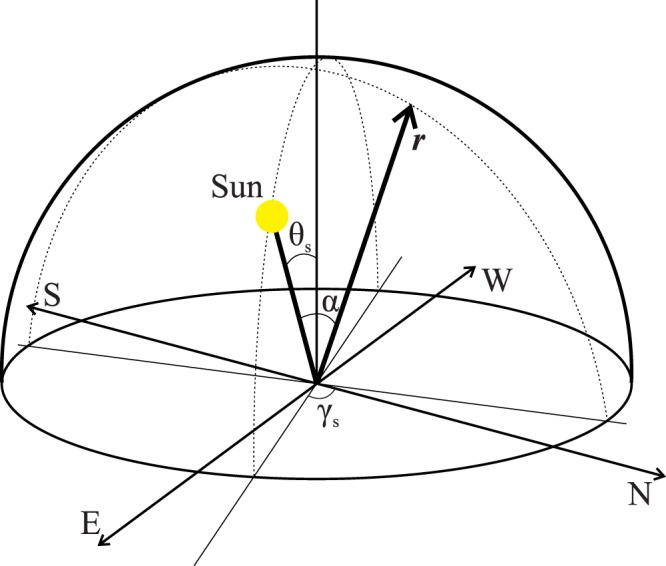
Diagram of the geometry used in the Rayleigh Sky Model. The vector ***r*** represents the viewing direction, γ_s_ is the solar azimuth angle and α is the scattering angle. The observer is at the origin.

The polarisation pattern of skylight is described by the Rayleigh Sky Model, which predicts the degree and angle of polarisation in a purely Rayleigh scattering atmosphere^50^. Figure 7 displays the geometry used in this model. Here the observer is located at the origin, looking in the direction given by the vector ***r***. The sun’s location in the sky is given by the solar zenith angle, θ_s,_ and azimuth, γ_s_. The angle α is the scattering angle, defined as the angle between the viewing and solar position vectors. The degree of linear polarisation, δ, is given by

2 $${\rm{\delta }}={{\rm{\delta }}}_{{\rm{\max }}}({\sin }^{{\rm{2}}}({\rm{\alpha }}))/({\rm{1}}+{\cos }^{{\rm{2}}}({\rm{\alpha }}))$$

The adjustment factor δ _max_ corrects for deviations from a perfect Rayleigh atmosphere, such as reflections from the Earth’s surface or multiple scattering within the atmosphere. The polarisation angle is orthogonal to the scattering plane, defined as the observer-solar-scattering point plane.

### Optical Flow

An optical flow algorithm was used to investigate the dynamics of the ash plume. Such algorithms are often applied to SO_2_ camera data to calculate the flow velocity of the SO_2_ plume^36,51–53^. We used the Farnebäck algorithm^54^ from the python OpenCV library, which allows for the calculation of dense flow rather than just sparse features. This algorithm tracks the movement of features from one frame to another, allowing a flow-vector for each pixel to be produced.

To measure the flow speed of the plume we applied a mask to the calculated flow-field to only include pixels where ash is present. Two regions were selected to investigate – the main plume and the downwind area where settling was observed (Fig. 3). An average vertical flow speed was found by taking the mean vertical component of the flow vectors in these regions.

## Electronic supplementary material

Supplementary Video S1

Supplementary Figure S1

## Data Availability

The datasets generated during and/or analysed during this study are available from the corresponding author on reasonable request.

## References

[1] Dingwell DB, Lavallée Y, Kueppers U (2012). Volcanic ash: A primary agent in the Earth system. Phys. Chem. Earth.

[2] Horwell CJ, Baxter PJ (2006). The respiratory health hazards of volcanic ash: A review for volcanic risk mitigation. Bull. Volcanol..

[3] Spence, R. J., Pomonis, A., Baxter, P. J., Coburn, A. W. & White, M. Building damage caused by the Mount Pinatubo eruption of June 15, 1991 In *Fire and Mud: eruptions and lahars of Mount Pinatubo, Philippines*. (eds Newall, C. G. & Punongbayan, R.) 1055–1061 (University of Washington Press, 1996).

[4] Bebbington M, Cronin SJ, Chapman I, Turner MB (2008). Quantifying volcanic ash fall hazard to electricity infrastructure. J. Volcanol. Geotherm. Res..

[5] Wilson TM (2012). Volcanic ash impacts on critical infrastructure. Phys. Chem. Earth.

[6] Chen WR, Zhao LR (2015). Review – Volcanic Ash and its Influence on Aircraft Engine Components. Procedia Eng..

[7] Langmann B, Folch A, Hensch M, Matthias V (2012). Volcanic ash over Europe during the eruption of Eyjafjallajökull on Iceland, April–May 2010. Atmos. Environ..

[8] Bonadonna Costanza, Costa Antonio, Folch Arnau, Koyaguchi Takehiro (2015). Tephra Dispersal and Sedimentation. The Encyclopedia of Volcanoes.

[9] Brown RJ, Bonadonna C, Durant AJ (2012). A review of volcanic ash aggregation. Phys. Chem. Earth.

[10] Mueller SB (2017). Stability of volcanic ash aggregates and break-up processes. Sci. Rep..

[11] Poret M, Costa A, Folch A, Martí A (2017). Modelling tephra dispersal and ash aggregation: The 26th April 1979 eruption, La Soufrière St. Vincent. J. Volcanol. Geotherm. Res..

[12] Prata AJ (1989). Infrared Radiative Transfer Calculations for Volcanic Ash Clouds. Geophsical Res. Lett..

[13] Prata AJ, Bernardo C (2009). Retrieval of volcanic ash particle size, mass and optical depth from a ground-based thermal infrared camera. J. Volcanol. Geotherm. Res..

[14] Lopez T (2014). Volcanic plume characteristics determined using an infrared imaging camera. J. Volcanol. Geotherm. Res..

[15] Harris DM, Rose WI (1983). Estimating Particle Sizes, Concentrations, and Total Mass of Ash in Volcanic Clouds Using Weather Radar. J. Geophys. Res..

[16] Lacasse C (2004). Weather radar observations of the Hekla 2000 eruption cloud, Iceland. Bull. Volcanol..

[17] Antuña JC (1996). Lidar measurements of stratospheric aerosols from Mount Pinatubo at Camaguey, Cuba. Atmos. Environ..

[18] Balis D (2016). Validation of ash optical depth and layer height retrieved from passive satellite sensors using EARLINET and airborne lidar data: the case of the Eyjafjallajökull eruption. Atmos. Chem. Phys..

[19] Lamb, O. D., De Angelis, S. & Lavallée, Y. Using infrasound to constrain ash plume rise. *J. Appl. Volcanol*. 10.1186/s13617-015-0038-6 (2015).

[20] Angelis S. De, Lamb O. D., Lamur A., Hornby A. J., von Aulock F. W., Chigna G., Lavallée Y., Rietbrock A. (2016). Characterization of moderate ash-and-gas explosions at Santiaguito volcano, Guatemala, from infrasound waveform inversion and thermal infrared measurements. Geophysical Research Letters.

[21] Carn S.A., Krotkov N.A. (2016). Ultraviolet Satellite Measurements of Volcanic Ash. Volcanic Ash.

[22] Yamamoto H, Watson IM, Phillips JC, Bluth GJ (2008). Rise dynamics and relative ash distribution in vulcanian eruption plumes at Santiaguito Volcano, Guatemala, revealed using an ultraviolet imaging camera. Geophys. Res. Lett..

[23] Tamburello G, Aiuppa A, Kantzas EP, McGonigle AJS, Ripepe M (2012). Passive vs. active degassing modes at an open-vent volcano (Stromboli, Italy). Earth Planet. Sci. Lett..

[24] Prata AJ (2014). Measuring SO2 ship emissions with an ultraviolet imaging camera. Atmos. Meas. Tech..

[25] Liu EJ, Cashman KV, Rust AC (2015). Optimising shape analysis to quantify volcanic ash morphology. GeoResJ.

[26] Krotkov NA (1999). Effect of particle non-sphericity on satellite monitoring of drifting volcanic ash clouds. J. Quant. Spectrosc. Radiat. Transf..

[27] Bluth GJS, Shannon JM, Watson IM, Prata AJ, Realmuto VJ (2007). Development of an ultra-violet digital camera for volcanic SO2 imaging. J. Volcanol. Geotherm. Res..

[28] Mori T, Burton M (2006). The SO2 camera: A simple, fast and cheap method for ground-based imaging of SO2 in volcanic plumes. Geophys. Res. Lett..

[29] Mori T, Burton M (2009). Quantification of the gas mass emitted during single explosions on Stromboli with the SO2 imaging camera. J. Volcanol. Geotherm. Res..

[30] Kern C (2015). Intercomparison of SO2 camera systems for imaging volcanic gas plumes. J. Volcanol. Geotherm. Res..

[31] Bennett EHS, Rose WI, Conway FM (1992). Santa María, Guatemala: A decade volcano. Eos, Trans. Am. Geophys. Union.

[32] ROSE WILLIAM I. (1972). Santiaguito Volcanic Dome, Guatemala. Geological Society of America Bulletin.

[33] Johnson JB, Lyons JJ, Andrews BJ, Lees JM (2014). Explosive dome eruptions modulated by periodic gas-driven inflation. Geophys. Res. Lett..

[34] Lavallée Y (2015). Thermal vesiculation during volcanic eruptions. Nature.

[35] Scharff L, Hort M, Gerst A (2014). The dynamics of the dome at Santiaguito volcano, Guatemala. Geophys. J. Int..

[36] Peters N, Hoffmann A, Barnie T, Herzog M, Oppenheimer C (2015). Use of motion estimation algorithms for improved flux measurements using SO2 cameras. J. Volcanol. Geotherm. Res..

[37] Sahetapy-Engel ST, Harris AJL (2009). Thermal-image-derived dynamics of vertical ash plumes at Santiaguito volcano, Guatemala. Bull. Volcanol..

[38] Manzella I, Bonadonna C, Phillips JC, Monnard H (2015). The role of gravitational instabilities in deposition of volcanic ash. Geology.

[39] Bonadonna C, Ernst GGJ, Sparks RSJ (1998). Thickness variations and volume estimates of tephra fall deposits: the importance of particle Reynolds number. J. Volcanol. Geotherm. Res..

[40] Kern C (2014). An automated SO2 camera system for continuous, real-time monitoring of gas emissions from Kīlauea Volcano’s summit Overlook Crater. J. Volcanol. Geotherm. Res..

[41] Burton MR (2014). SO2 flux monitoring at Stromboli with the new permanent INGV SO2 camera system: A comparison with the FLAME network and seismological data. J. Volcanol. Geotherm. Res..

[42] Scollo, S., Bonadonna, C. & Manzella, I. Settling-driven gravitational instabilities associated with volcanic clouds: new insights from experimental investigations. *Bull. Volcanol*. 1–14, 10.1007/s00445-017-1124-x (2017).

[43] Schotland RM, Sassen K, Stone R (1971). Observations by Lidar of Linear Depolarization Ratios for Hydrometeors. Journal of Applied Meteorology.

[44] Groß S (2012). Dual-wavelength linear depolarization ratio of volcanic aerosols: Lidar measurements of the Eyjafjallajökull plume over Maisach, Germany. Atmos. Environ..

[45] Pisani G (2012). Lidar depolarization measurement of fresh volcanic ash from Mt. Etna, Italy. Atmos. Environ..

[46] Scollo S (2012). Monitoring Etna volcanic plumes using a scanning LiDAR. Bull. Volcanol..

[47] Sun W (2013). For the depolarization of linearly polarized light by smoke particles. J. Quant. Spectrosc. Radiat. Transf..

[48] Labhart T, Meyer EP (2002). Neural mechanisms in insect navigation: Polarization compass and odometer. Curr. Opin. Neurobiol..

[49] Horvath G (2011). On the trail of Vikings with polarized skylight: experimental study of the atmospheric optical prerequisites allowing polarimetric navigation by Viking seafarers. Philos. Trans. R. Soc. B Biol. Sci..

[50] Suhai B, Horváth G (2004). How well does the Rayleigh model describe the E-vector distribution of skylight in clear and cloudy conditions? A full-sky polarimetric study. J. Opt. Soc. Am. A. Opt. Image Sci. Vis..

[51] Gliß J (2017). A Python Software Toolbox for the Analysis of SO2 Camera Data. Implications in Geosciences. Geosciences.

[52] Gliß J, Stebel K, Kylling A, Sudbø A (2018). Improved optical flow velocity analysis in SO2 camera images of volcanic plumes - Implications for emission-rate retrievals investigated at Mt Etna, Italy and Guallatiri, Chile. Atmos. Meas. Tech..

[53] Peters N, Oppenheimer C (2018). Plumetrack: Flux calculation software for UV cameras. Comput. Geosci..

[54] Farnebäck, G. *In Lecture Notes In Computer Science* (eds Bigun, J. & Gustavsson, T.) **2749**, 363–370 (Springer, 2003).

