# Supplementary material for: Quantification of ash sedimentation dynamics through depolarisation imaging with AshCam
Source: Sci Rep. 2018 Oct 24;8:15680. doi: 10.1038/s41598-018-34110-6 (PMC6200762; doi:10.1038/s41598-018-34110-6)
Supplement: Supplementary file 2 — Supplementary Figure S1 [file 41598_2018_34110_MOESM2_ESM.docx]

# Quantification of ash sedimentation dynamics through depolarisation imaging with AshCam

Ben Esse* ^1^, Michael Burton ^1^, Matthew Varnam ^1^, Ryunosuke Kazahaya ^2,1^, Paul A. Wallace ^3^, Felix Von-Aulock^3^, Yan Lavallée ^3^, Giuseppe Salerno ^4^, Simona Scollo ^4^, Hugh Coe ^1^

^1^ School of Earth and Environmental Sciences, The University of Manchester, Manchester, M13 9PL, UK

^2^ Geological Survey of Japan, National Institute of Advanced Industrial Science and Technology, Tsukuba, Japan

^3^ Department of Earth, Ocean and Ecological Sciences, University of Liverpool, Liverpool, L69 3GP, UK

^4^ Istituto Nazionale di Geofisica e Vulcanologia, Osservatorio Etneo, sezione di Catania, Piazza Roma 2, 95123, Catania, Italy

Corresponding author: Ben Esse

Email: [benjamin.esse@manchester.ac.uk](mailto:benjamin.esse@manchester.ac.uk)


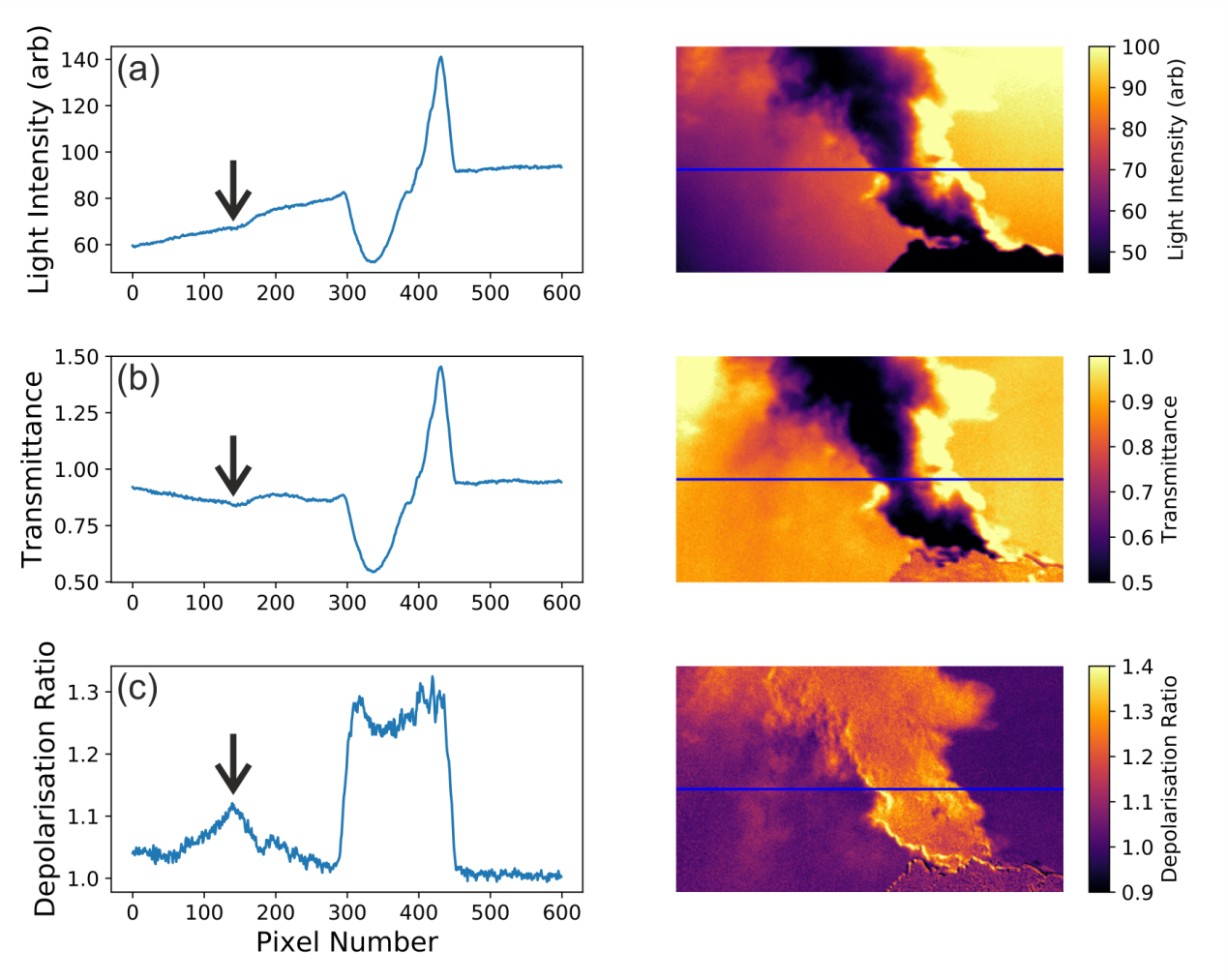


**Supplementary Figure S1: Images and cross-sections of the plume of Santiaguito 260 s after the onset of the explosion at different analysis stages.** (a) Dark corrected intensity image from a single camera. (b) Dark corrected transmittance image, formed by dividing the plume image by the reference taken before the explosion. Transmittance values higher than 1 are caused by reflections from the surface of the plume. (c) Depolarisation ratio image. The graphs show the cross-section given by the blue line in the images (averaged across 10 pixels vertically). The signal from the ash is much clearer in the depolarisation ratio than in either the normal intensity or transmittance images.
